# HTLV infection and cessation of breastfeeding: context and challenges in implementing universal prevention policies in Brazil

**DOI:** 10.1590/S2237-96222023000200025

**Published:** 2023-09-18

**Authors:** Carolina Rosadas, Angélica Espinosa Miranda

**Affiliations:** 1 Section of Virology, Department of Infectious Disease, Imperial College London, London, United Kingdom; 2 Ministério da Saúde do Brasil, Secretaria de Vigilância em Saúde e Ambiente, Brasília, DF, Brazil

The benefits of breastfeeding are extensive and well known. However, in certain medical conditions it is contraindicated, such as in women living with a human T-cell lymphotropic virus type 1 (HTLV-1) infection.^1^ HTLV-1 was discovered in the 1980s, although its impact was underestimated and neglected for many years.^2^ It is commonly reported in the scientific literature that only 5% to 10% of infected individuals experience symptoms of the virus infection; nevertheless, there is a consensus that its deleterious effects are broader and more frequent than previously recognized.^2^ HTLV-1 is the etiological agent of adult T-cell leukemia/lymphoma (ATLL), an unfavorable prognosis neoplasm, and HTLV-1-associated myelopathy (HAM), a progressive neurological disease. The virus can also cause inflammatory diseases, such as uveitis, infective dermatitis, encephalitis, as well as pulmonary, intestinal and urinary alterations, in addition to erectile dysfunction, psychological disorders and negative impact on co-infections.^3^ A study commissioned by the World Health Organization (WHO) revealed that the risk of death among people living with HTLV-1 increases by 57%.^4^ Notwithstanding, the virus, which affects between 800,000 and 2.5 million Brazilians, remains neglected in the country.

HTLV-1 is transmitted through unprotected sexual intercourse, contact with infected blood (blood transfusion, organ transplantation or contact with sharp objects) and mother-to-child transmission, mainly through breastfeeding. There is no vaccine or curative treatment for HTLV-1.^3^ ^),(^ ^5^ In Brazil, the risk of parenteral transmission is reduced due to universal screening of blood and organ donors.^6^ ^),(^ ^7^ In order to prevent mother-to-child transmission, the Brazilian Ministry of Health recommends the cessation of breastfeeding for mothers living with the virus.^8^ ^),(^ ^9^ Antiretroviral therapy is not effective in reducing HTLV-1 proviral load and therefore it is not recommended for people living with HTLV-1.^10^ Avoidance of breastfeeding, on the other hand, prevents about 85% of childhood infections, consequently it is considered the most effective intervention currently available.^11^

The public policy proposed by the Ministry of Health (lactation inhibition) is also a strategy recommended in several countries, such as Japan, Chile, Colombia, Uruguay, Santa Lucia and Canada, as well as agencies, such as the National Institute of Health in the United States^11^ and the Brazilian Federation of Gynecology and Obstetrics Associations (Federação Brasileira de Ginecologia e Obstetrícia - FEBRASGO). Contraindication to breastfeeding is included in the Clinical Protocol and Therapeutic Guidelines for prevention of Mother-to-child Transmission and Sexually Transmitted Infections (STIs).^8^ ^),(^ ^9^ However, there is still a lack of knowledge among health professionals and policymakers on HTLV-1 and infection prevention measures.^10^ ^),(^ ^12^

**Figure 1:**
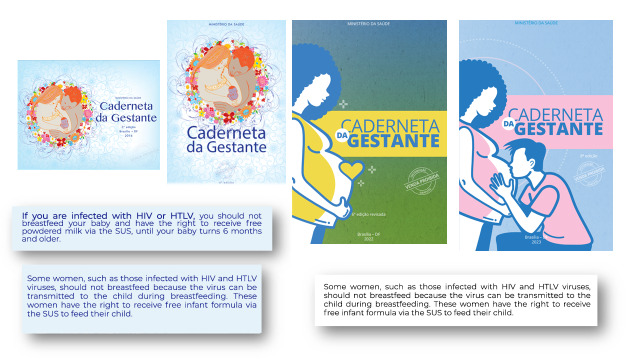
Pregnant Woman's Booklet, published by the Brazilian Ministry of Health, indicating the right to infant formula for mothers living with HTLV-1

In the Brazilian context, the right to free infant formula provided by the Brazilian National Health System (Sistema Único de Saúde - SUS), stands out. Information Note No. 4/2021-CGIST/DCCI/SVS/MS addresses the recommendation for the use of cabergoline in lactation inhibition and infant formula in the prevention of mother-to-child transmission of HIV and HTLV.^13^ Despite the fact that it has been included in the Pregnant Woman's Booklet since its third edition, in 2016, (Figure 1), many people are unaware of their right to infant formula.^14^ Thus, many barriers persist in the access to the recommended public policies.

A = Pregnant Women's Booklet, 2016 edition; B = Pregnant Women's Booklet, 2018 edition; C = Pregnant Women's Booklet, 2022 edition; D = Pregnant Women's Booklet, 2023 edition; E = Information on HTLV, found in the 2016 and 2018 editions; F = Information on HTLV-1, found in the 2022 and 2023 editions.

Source: Ministry of Health, Brazil, Pregnant Women's Booklet 2016, 2018, 2022, 2023 editions. Available at: https://aps.saude.gov.br/biblioteca/visualizar/MjE2NQ==, https://bvsms.saude.gov.br/bvs/publicacoes/caderneta_gestante_versao_eletronica_2022.pdf.

Note: HTLV-1 = Human T-cell lymphotropic virus type 1.

Recently, the WHO has recognized HTLV-1 as a public health problem.^5^ Concurrently, the Pan American Health Organization (PAHO) has been focusing on HTLV-1 and acknowledging its significance in the Americas region; the prevention of mother-to-child transmission is considered a priority action in response to the virus, both by PAHO and WHO, and has been addressed in the WHO’s strategic plan for STIs.^15^ ^)-(^ ^17^ The inclusion of HTLV-1 in the initiative to eliminate mother-to-child transmission of HIV, syphilis, hepatitis and Chagas disease is currently under discussion by international agencies.^17^ Brazil plays a prominent role in responding to HTLV-1, however, despite this international recognition,^16^ it still has incipient policies that require expansion and strengthening.

**Figure 2:**
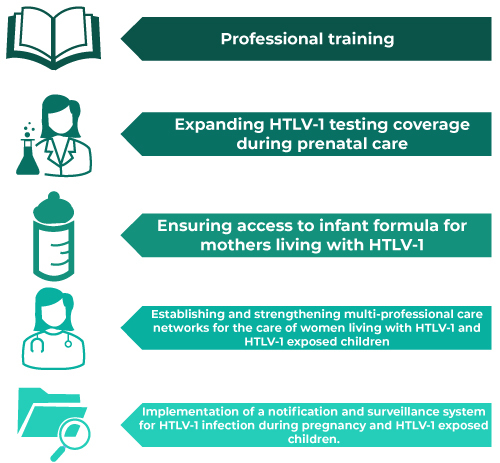
Key aspects for expanding and strengthening the response to HTLV-1 in the context of maternal and child health

Note: HTLV-1 = Human T-cell lymphotropic virus type 1.

Among the priority actions to strengthen the response to HTLV-1 in the context of maternal and child health in the country, the following stand out: (i) intensification of capacity building for healthcare professionals and public policymakers; (ii) expanding HTLV-1 testing coverage in antenatal care; (iii) ensuring access to infant formula; (iv) establishing and strengthening multidisciplinary care networks for the comprehensive support and clinical follow-up of pregnant women living with HTLV-1 and HTLV-1 exposed children; and (v) implementing a surveillance system for HTLV-1 infection in pregnant women and exposed children (Figure 2).

The sensitization of managers is of fundamental importance for the inclusion of HTLV-1 in the public health agenda, regarding capacity building for professionals, especially in primary healthcare. This is a crucial action to ensure that mothers have access to adequate information about HTLV-1, modes of transmission, associated diseases, and methods for reducing transmission risks. The Ministry of Health has included HTLV-1 in a training course on STIs and regularly conducts awareness campaigns.^12^ Continuous work focusing on this subject is essential, as well as stronger collaboration with important stakeholders such as medical and nursing councils, and professional associations such as FEBRASGO and the Brazilian Society of Pediatrics. Collaboration with the Ministry of Education, for the inclusion of HTLV-1 in the curriculum of professional development courses, is another strategy to be take into consideration. In addition, the coordination between the Ministry of Health and the Ministry of Women is valuable due to their shared interest in this topic.

The identification of mothers living with HTLV-1 is essential for the success of any strategy aimed at reducing mother-to-child transmission of the virus. However, despite its cost-effectiveness, testing for HTLV-1 during antenatal care^18^ is not universally offered in Brazil, and its evaluation by the National Committee for Health Technology Incorporation in the Brazilian National Health System (Comissão Nacional de Incorporação de Tecnologias - CONITEC) is currently underway, at the time of the conclusion of this article. It is noteworthy that antenatal screening had been included in Ordinance GM/MS No. 715, issued on April 4, 2022, when the now defunct maternal and child healthcare program (rede de atenção materno-infantil - RAMI) was established,^19^ and was repealed by Ordinance GM/MS No. 13, on January 13, 2023.^20^ The diagnosis of HTLV-1 involves both screening and confirmatory tests, all of which are included in the SUS.^3^ Some Federative Units, such as Bahia,^21^ Mato Grosso do Sul and the Federal District, as well as municipalities such as Belo Horizonte (state of Minas Gerais) and Rio Verde (state of Goiás), have already issued ordinances guaranteeing the implementation of universal testing of pregnant women. Recently, other state health departments have established technical cooperation in the implementation HTLV-1 line of care network. Important measures for expanding HTLV-1 testing coverage include technical training [with a particular focus on professionals from the Central Public Health Laboratories (Laboratórios Centrais de Saúde Pública - LACEN)], promoting interstate discussions, developing informative notes, manuals and technical guides, in addition to allocating financial resources. In this sense, the support of the federal government can act as a catalyst.

It is worth highlighting the need for an acceptable, feasible, affordable, sustainable and safe (AFASS) intervention. Although there is limited data, the literature demonstrates that prenatal testing and intervention have high acceptance rates: over 90% of mothers choose to follow medical recommendations, a finding confirmed by representatives of people living with HTLV-1, both in Brazil and in other countries.^11^ ^),(^ ^16^ ^),(^ ^22^ In order for the intervention to be AFASS in Brazil, access to free infant formula is essential. People living in vulnerable situations, with low income and low education level, are the most commonly affected by HTLV-1. Therefore, it is crucial that women living with this virus receive support, have access to adequate and understandable information about HTLV-1, prevention and care measures, and have their rights guaranteed (including the supply of infant formula). Access to infant formula should be immediate. Maternity hospitals must provide a sufficient quantity of infant formula until the HTLV-1 exposed child is properly enrolled in follow-up services.^13^ Clear guidance on the hygienic and safe preparation, storage and feeding of breast-milk substitutes needs to be provided. It is important to ensure that the family has access to potable water and items such as baby bottles and cleaning products. Health professionals should take into consideration that stigma can be a barrier to adherence and, therefore, should focus on providing support and acceptance.^23^ In a context that promotes breastfeeding, those mothers who do not breastfeed may feel excluded and pressured. Several initiatives observed in Bahia, a state with a comprehensive care network for HTLV-1, aim to support mothers living with HTLV-1.^21^ ^),(^ ^24^

The establishment of multi-professional care networks for HTLV-1 is another priority. A survey conducted by the Ministry of Health showed that centers for HTLV care are scarce and unevenly distributed in the country. Many of these centers are associated with research groups, without government financial support.^12^ It is necessary to reverse this situation in order to ensure universal and equitable access to healthcare services.

Implementation of a notification and surveillance system for the infection in pregnant women and exposed children is important for identifying priority areas, designing more effective interventions and monitoring the impact of existing policies.^10^ ^),(^ ^12^ The laboratory interface and greater coordination with the Digital Health and Information Secretariat of the Ministry of Health will be welcome, as well as the implementation of the dialogue with the state and municipal health departments, so that the three spheres of government are involved in this process.

Cessation of breastfeeding, an effective measure to prevent mother-to-child transmission of HTLV-1, is recommended by the Brazilian Ministry of Health. It is crucial to ensure (i) HTLV-1 testing during antenatal care and (ii) an AFASS intervention. Access to free infant formula plays an important role in promoting adherence; however, many mothers living with the human T-cell lymphotropic virus type 1 - HTLV-1 are unaware of this right. Despite the advancements achieved, it is necessary to expand and strengthen public policies aimed at controlling this important, yet neglected disease.
